# One-Off Focused Teaching Can Improve Trainee Confidence, Knowledge and Skillset in Understanding and Therapeutically Engaging People With a Diagnosis of Personality Disorder

**DOI:** 10.1192/bjo.2024.304

**Published:** 2024-08-01

**Authors:** Sil-Jun Lau, Daniel Meek

**Affiliations:** South London and Maudsley NHS Foundation Trust, London, United Kingdom

## Abstract

**Aims:**

To design, deliver and evaluate teaching for psychiatry trainees on personality disorder (PD) with the following objectives: to promote understanding and empathy for people with a diagnosis of PD; to equip trainees with skills they can immediately use for therapeutically engaging patients with PD; to introduce the evidence-based treatments that underpin these techniques; and to increase confidence in offering therapeutic clinical encounters for patients with PD.

**Methods:**

A single teaching session was designed and delivered to core psychiatry trainees in three components. First, an interactive lecture was delivered on the theory of personality disorder as understood by two evidence-based psychotherapies: Mentalization-Based Treatment (MBT) and Transference-Focused Psychotherapy (TFP). Second, techniques from both were introduced as skills they can readily apply to clinical practice. Lastly, role-play scenarios with original scripts were worked through to highlight theory and techniques. Evaluation was conducted through anonymous participant-rated scores matched to learning objectives pre- and post-delivery of teaching.

**Results:**

20 participants (n = 20) completed the evaluation. 90% of respondents agreed/strongly agreed that they frequently encountered patients with PD. There was high pre-existing confidence in recognising PD in clinical practice; this was little changed by the teaching. Before the teaching, 45% of respondents agreed/strongly agreed with the statement saying they are confident offering clinical encounters for patients with PD; this changed to 90% post-delivery. Pre-delivery, 45% agreed/strongly agreed they possessed skills they could use clinically for PD; this increased to 75% post-delivery. Pre-delivery, 60% agreed/strongly agreed that they can generally empathise with people with PD; this increased to 90% post-delivery. Self-rated knowledge of evidence-based treatments for PD increased for both MBT (20% pre-delivery to 85% post-delivery) and TFP (15% to 75%). 95% of respondents agreed/strongly agreed that they will try out new skills learnt from the session. 100% of respondents agreed/strongly agreed that the teaching was overall useful.

**Conclusion:**

This study shows it is possible to make positive effects on trainee confidence, knowledge and skill in relation to PD in a short and one-off timeframe. Future efforts should include attempts to replicate these findings on larger numbers of participants, across different training and non-training medical grades and in non-medical staff. Future evaluation should also observe if positive changes are sustained across time or lead to improvements in clinical outcomes and patient satisfaction.

